# Advanced preclinical functional magnetic resonance imaging of the brain

**DOI:** 10.1038/s44303-025-00085-z

**Published:** 2025-06-18

**Authors:** Jan Klohs, Way Cherng Chen, Rikita Araki

**Affiliations:** 1 Bruker BioSpin GmbH & Co. KG, Ettlingen, Germany; 2 Bruker Singapore Pte. Ltd, Singapore, Singapore; 3 Bruker Japan K.K, Yokohama, Japan

**Keywords:** Imaging techniques, Functional magnetic resonance imaging, Magnetic resonance imaging

## Abstract

Functional magnetic resonance imaging (fMRI), exploiting the blood oxygen level-dependent (BOLD) contrast, is the most widely used technique to study brain function. Combined with tools from biotechnology, molecular biology, and genetics, preclinical fMRI offers unparalleled opportunities to experimentally test causal hypotheses that are beyond the reach of human research. Here, we review recent progress in MRI hardware development, provide recommendations for BOLD fMRI protocol optimization, and discuss recent applications.

## Introduction

### The unique role of preclinical MRI for functional brain studies

**Fig. 1:**
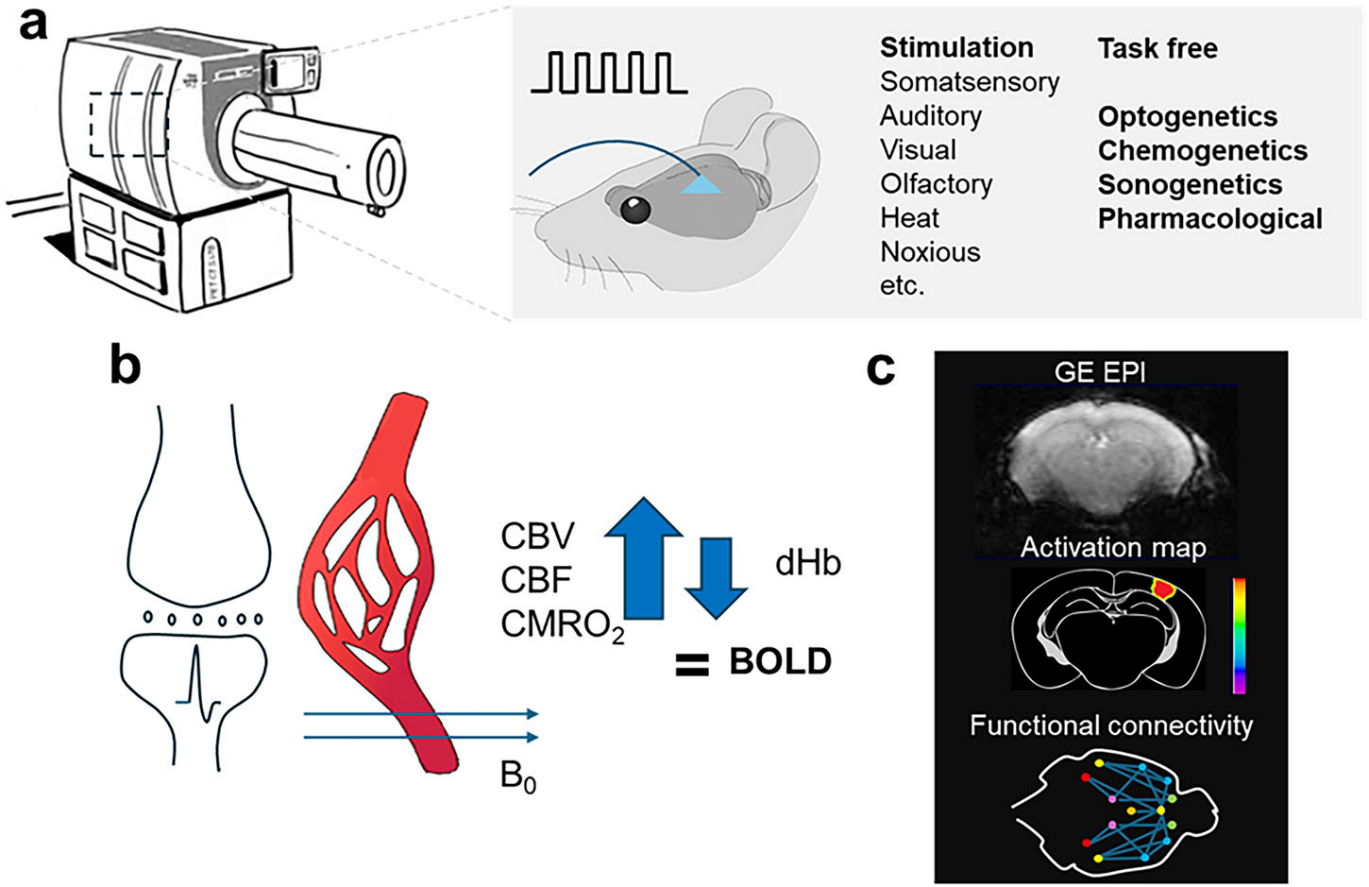
Components of preclinical fMRI. **a** MRI hardware geared for the physiological imaging of different animal species. Data is acquired under task-free conditions, using different types of stimulation, or using optogenetic, chemogenetic, sonogenetic, or pharmacological interventions. **b** In fMRI, blood oxygen level dependent (BOLD) contrast is generated when increased local neuronal activity results in an increase in cerebral blood volume (CBV), cerebral blood flow (CBF), and cerebral metabolic rate of oxygen (CMRO_2_). As the hemodynamic response exceeds the increase in oxygen consumption, there is a relative decrease in paramagnetic deoxyhemoglobin (dHb) concentration compared to diamagnetic oxyhemoglobin in the blood vessels. This change in dHb ultimately leads to the recordable BOLD signal. **c** BOLD signal is recorded using a T_2_*-weighted GE EPI sequence that can be transformed into maps of focal brain activity and functional connectivity. GE EPI data were acquired for educational purposes using a BioSpec Maxwell 94/17. All Bruker in vivo animal work was approved by the institutional animal care and use committee (IACUC) and local authorities and conducted under a valid study permit (Germany AZ 123456).

Functional neuroimaging aims to understand how brain activity relates to cognition and behavior, how it changes during development and aging, and how it is altered in disease states. The most widely used technique to date is functional magnetic resonance imaging (fMRI), which exploits the blood oxygen level dependent (BOLD) contrast^1^. The method has become a workhorse in neuroscience studies, as well as in psychiatric and neurological research. The approach is directly translatable and its use in a variety of animal species has been demonstrated, including rodents, fish, birds, and non-human primates (NHP) (Fig. 1).

Biotechnology, molecular biology, and genetics provide preclinical fMRI scientists with tools to experimentally test causal hypotheses that are often not attainable in human research. Genetic alterations via the activation of genes or expression of foreign genes in specific cells and tissues can be linked to changes in brain function and connectivity, and provide models of human brain disorders^2,3^. In addition, interventional systems using either virally inducing light-sensitive channels with light stimulation (optogenetics), chemically engineered receptors and exogenous molecules specific for those receptors (chemogenetics), or mechanosensitive ion channels with ultrasound (sonogenetics) can alter the activity of specific brain cells and circuits in the living animal and thus enable mapping of functional circuitry and the study of the role of specific cells or brain regions^4–7^. Lastly, the ability to perform transcriptome, proteome, histological, and immunohistochemical analyses allows linking brain function to its cellular and molecular underpinnings.

BOLD fMRI is most often performed with a T_2_*-weighted gradient echo (GE) planar imaging (EPI) sequence, which can record BOLD signals with subsecond time resolution. However, EPI sequences put high demands on scanner hardware. Dedicated equipment for handling animals and performing experimental testing is required for preclinical fMRI. While experimental paradigms, computational tools and standardization issues are discussed elsewhere^8–12^, MRI hardware and protocol considerations have received little attention. Here, we will review MRI hardware and EPI method and protocol optimization for preclinical fMRI experiments and showcase their applications.

### Probing a weak hemodynamic response at high and ultrahigh field

In BOLD fMRI, contrast is generated when increased local neuronal activity results in an increase in oxygen extraction, cerebral blood flow, and cerebral blood volume. As the hemodynamic response exceeds the increase in oxygen consumption, there is a relative decrease in paramagnetic deoxyhemoglobin concentration compared to diamagnetic oxyhemoglobin in the blood vessels^1^. This produces a weak transient rise in the signal when using a T_2_*-weighted EPI sequence for detection. The detection sensitivity of fMRI acquisitions is described by the functional contrast-to-noise ratio (${fCNR}$) per unit time in Eq. (1).

1 $${fCNR}=\left(\frac{\varDelta S}{S}\right)\left({tSNR}\right)$$

where $\frac{\triangle S}{S}$ is the evoked fractional BOLD signal change and ${tSNR}$ is the temporal signal-to-noise ratio (SNR), defined as the mean signal amplitude divided by the standard deviation of the detrended fMRI time series^13^.

Preclinical fMRI acquisitions put high demand on the ${fCNR}$ because: (i) the $\frac{\varDelta S}{S}$ only exceeds the baseline signal fluctuation by a few percent^1^; (ii) the dimensions of animal brains require a high spatial resolution to precisely map the functional responses; and (iii) accurate mapping of the hemodynamic response function in stimulus-evoked fMRI requires a high temporal resolution^14^, decreasing ${tSNR}$ at high spatial resolution.

**Fig. 2:**
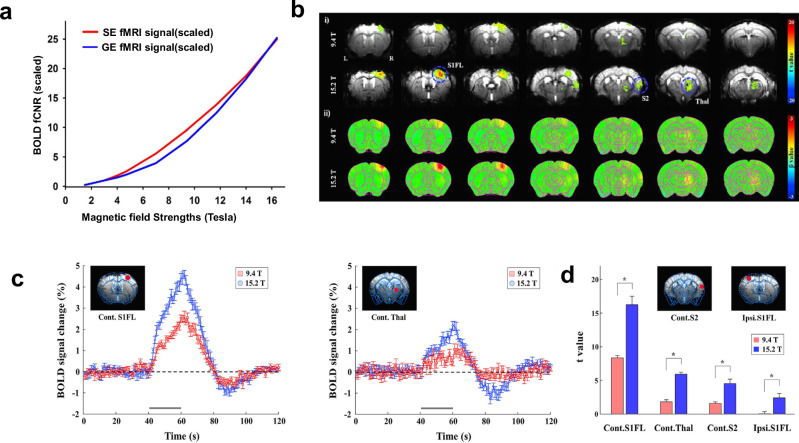
Magnetic field dependence of the BOLD functional contrast-to-noise ratio (fCNR). **a** Signal amplitude of micro-vasculature as a function of magnetic field strength for gradient echo (GE, blue) and spin echo (SE, red) EPI sequences, normalized to 3 T and scaled with B_0_^1.65^^15^. Taken with modifications from [Uludag and Blinder 2018]^77^. **b** Field strength-dependent (9.4 Tesla vs. 15.2 Tesla) BOLD response to 20-s forepaw stimulation in lightly anesthetized mice. Multi-slice fMRI maps of i) one representative animal overlaid on the original EPI image with a statistical threshold (uncorrected *p* < 0.001, cluster size >5 voxels) and ii) group-averaged analysis without statistical thresholds (*n* = 7, respectively) at each magnetic field. **c** fMRI time courses (average of seven animals) obtained from the region-of-interest of the contralateral S1FL and thalamus were plotted for 9.4 Tesla (red) and 15.2 Tesla (blue). Error bars, SEM; gray horizontal bar, 20-s stimulus duration. **d** Field strength-dependent statistical t values from four different ROIs of contralateral forelimb primary somatosensory area (Cont.S1FL), thalamus (Cont.Thal), secondary somatosensory area (Cont.S2), and ipsilateral primary somatosensory area (Ipsi.S1FL). error bars, SEM; *p < 0.05 (*n* = 7, independent *t* test). Taken from [Jung 2019]^18^.

Advances in the design of preclinical MRI systems, most notably the move to ultrahigh fields, allow for the weak $\frac{\triangle S}{S}$ response with a high ${fCNR}$ (Fig. 2). It has been experimentally demonstrated that the SNR increases with static main magnetic field B_0_^15^. However, physiological noise increases with B_0_ as well^16^, which implies that SNR gains above a certain level no longer translate into improved *tSNR*. Preclinical studies using an MR system operating at 3 Tesla have been reported, but most studies are conducted at 7 Tesla and 9.4 Tesla^9,11^. Beyond this, preclinical ultrahigh field MR systems ranging from 11.7 Tesla to 18 Tesla are available. They offer the advantage of a supra-linear increase in ${fCNR}$ with B_0_ due to a stronger BOLD contrast at ultrahigh fields^17,18^.

### High-performance gradient coils and amplifiers

The species used in fMRI experiments determines the required bore size of the magnet, which can range from 89 to 650 mm^19^. The inner dimension of the magnet bore plays a crucial role in the homogeneity of the magnetic field, with wider and longer bores being able to generate more homogeneous magnetic fields, but adding to the weight of the magnet and siting requirements.

The spatial and temporal resolution achievable in fMRI is highly dependent on the performance of the imaging gradients. High-performing gradient coils and gradient amplifier combinations not only need to reach the highest gradient strengths but also have high slew rates^20^. Modern preclinical MRI scanners have maximum gradient strengths of 400–1000 mT/m and slew rates of 1000–9000 T/m/s^21^. Gradient electronics also need to have excellent performance to ensure that the desired gradient pulse shapes are played out during image acquisition perfectly, and eddy current reduction is performed to the best. For this, gradient coils have dedicated structures to avoid eddy currents and several windings that do not produce any magnetic field outside of the coil. This is known as self-shielding or active shielding^20^. Precise digital pre-emphasis is another method that modulates the driving current for the gradient pulse to compensate for the residual eddy current, which would otherwise disturb the desired waveform^20^. To achieve high gradient duty cycles, it is important to efficiently dissipate the generated heat^22,23^. To this end, gradient systems contain pipes through which refrigerated water is circulated. Temperature sensors are mounted to control the temperature of the gradient systems. An inevitable consequence of rapid switching of the powerful gradients used in EPI is the generated acoustic noise^24^, which may be undesirable and require habituation and noise reduction measures in some circumstances.

### Dedicated radiofrequency coils to improve SNR

For fMRI studies, single-loop surface coils can be used in combination with an actively decoupled transmit volume resonator to achieve uniform tissue excitation and higher SNR (compared to a volume resonator transmit and receive setup) at the expense of distance-dependent coil sensitivity, resulting in inhomogeneous and reduced signal coverage^25,26^. Single-loop surface coils offer the advantage of granting access to the skull, e.g., for optogenetic stimulation, electrophysiology, or optical imaging equipment.

**Fig. 3:**
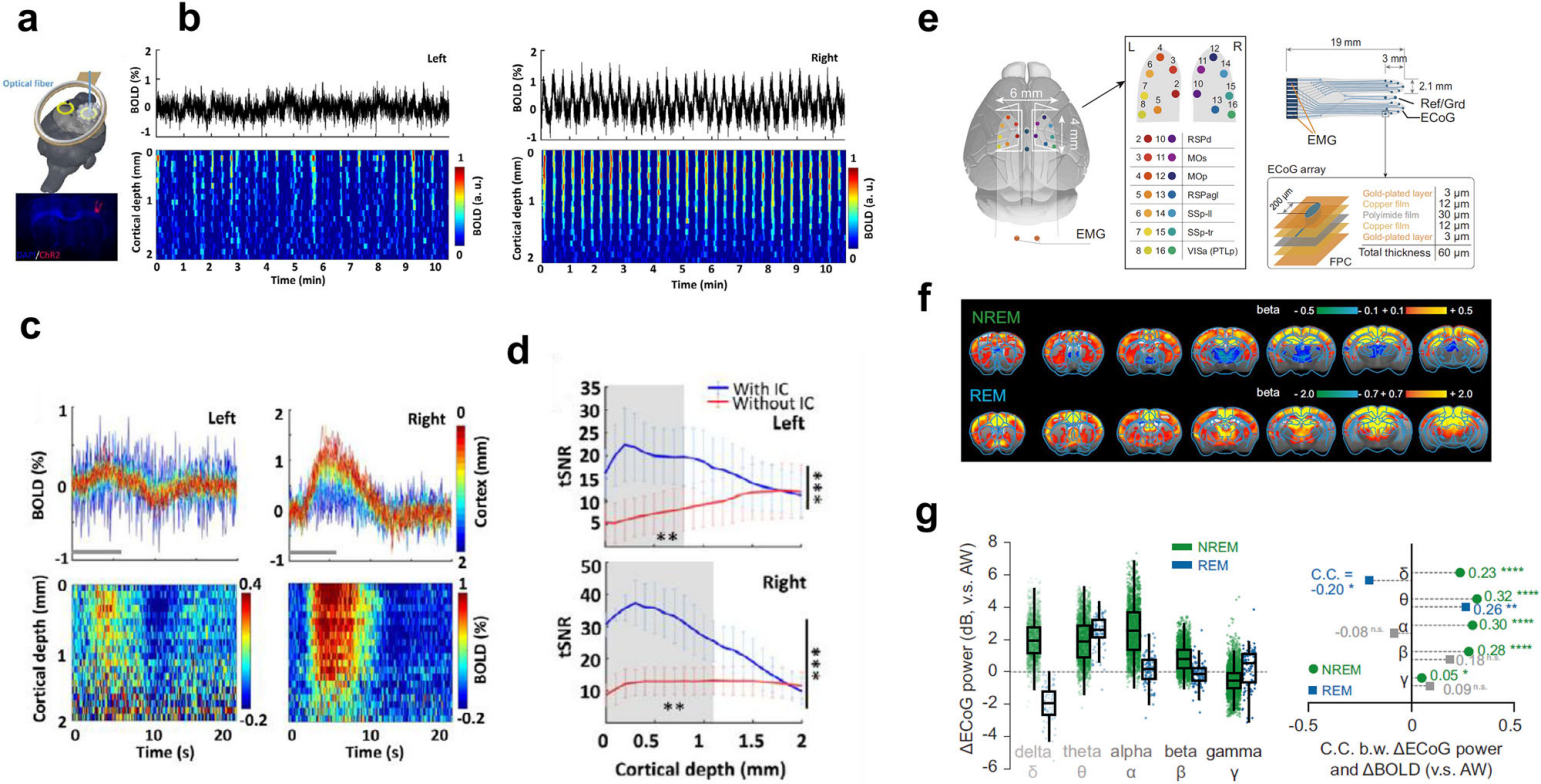
Implantable radiofrequency coils and MR-compatible electrophysiological recording in rodent fMRI. **a**–**d** Optogenetically evoked BOLD with implantable inductively coupled detector. **a** Schematic drawing of two inductive coils with an optical fiber inserted into the right forepaw somatosensory cortex (blue). A representative wide-field fluorescence image illustrates ChR2-mCherry expression. **b** Averaged time course (top) and normalized spatiotemporal map (bottom) BOLD responses induced directly by optogenetic stimulation in right forepaw somatosensory cortex (right) and projected left hemisphere (left). **c** BOLD percentage-change time courses (top) and maps (bottom) for each epoch in each voxel along cortical depth on both hemispheres (*n* = 3). Gray lines indicate light stimulation. **d** The comparison between both hemispheres shows significantly higher tSNR in images acquired with (blue) implanted inductive coils over images acquired without (red) implanted inductive coils (paired-sample t-test, ***p < 0.001, **p < 0.01 for the gray shadow, upper panel 0–0.8 mm, lower panel 0–1.1 mm, *n* = 3 rats, mean ± SD). The asterisks on the right side indicate a 3-fold sensitivity gain. Taken with modifications from [Chen 2022]^28^. **e**–**g** fMRI using MR-compatible electrophysiology recording in un-anesthetized mice. **e** Design and location of MR-compatible electrocorticogram (ECoG array). Two gold wires (orange) were inserted in the nuchal region for electromyography (EMG) recording. Right panel, the diagram of a multi-electrode ECoG array and its layered construction. FPC flexible printed circuit. **f** The setup was used to assess brain-wide BOLD activation of non-rapid eye movement (NREM) and rapid eye movement (REM) sleep in mice using simultaneous electrophysiology. Shown are group BOLD activation maps of NREM and REM compared to awake (AW) state (FDR corrected, *p* < 0.05; *n* = 46). **g** Relative ECoG band-limited power in NREM and REM compared to AW state and Pearson’s correlation coefficients (C.C.). The boxes show the first and third quartiles; inner line is the median over sessions; whiskers represent minimum and maximum values (outliers removed). delta (δ), 1–4 Hz; theta (θ), 5–10 Hz; alpha (α), 11–20 Hz; beta (β), 21–40 Hz; gamma (γ), 41–100 Hz. Statistical significance was calculated by two-tailed *t* test. *p < 0.05; **p < 0.01; ****p < 0.0001; n.s., no significance. Taken from [Yu 2023]^78^.

Multi-channel brain array coils are also commonly used for preclinical fMRI, since they allow wider coverage with the possibility of accelerated acquisition via parallel imaging and multiband imaging. Most commercially available surface array/ single loop coils are rigid and may not be easily placed at the closest proximity to the animal heads for the greatest sensitivity. More ergonomic surface coils have been designed to allow the coil elements to be as close to the organ of interest as possible. A stretchable receive coil at 7 Tesla reported a 40% increase in SNR and over 30% increase in coverage^27^. Implantable radiofrequency coils have been successfully used in rodents and NHPs to provide higher SNR due to their even closer proximity to the brain (Fig. 3)^28^. An implanted single-loop coil was reported to have a 100% improved SNR over a commercial 2 × 2 phase array coil, while an innovative figure 8 implanted loop coil showed a 500% increase in SNR^29^. However, the surgery for coil implantation may induce tissue damage and susceptibility image artifacts. Alternatively, an inductively coupled detector has been implanted on rat skulls to achieve localized fMRI signal enhancement of 2–3-fold^28^.

Cooling the RF coil assembly and preamplifier to cryogenic temperatures can tremendously increase SNR by reducing the electronic noise of the coil. Cryogenic quadrature and array radiofrequency coils using liquid nitrogen or cryogenic helium are available and have gained popularity in preclinical fMRI studies^9,12,30^. For example, when compared to a room temperature coil, a gain of ~3 in SNR and ~1.8 in ${tSNR}$ of the BOLD response was reported for a cryogenic coil used at 9.4 Tesla^31^.

### Studying brain function under physiological conditions

Animal handling and preparation is a critical aspect in preclinical fMRI studies to (i) safeguard the well-being of the animal under study; (ii) obtain functional brain states that are not or only minimally confounded by experimental conditions; and (iii) obtain EPI images of good quality and reproducibility. To this end, fMRI experiments are usually conducted in anesthetized animals, thereby reducing stress and motion. But anesthesia can be a confounder because it can impair neuronal transmission and hemodynamic function, which may directly alter the BOLD signal and/or affect animal physiology, such as respiration and cardiovascular function, which can modulate the BOLD signal^10^. fMRI experiments on awake animals have been successfully performed^30,32^. However, imaging awake animals requires special hardware setups and habituation of the animals to the experimental conditions.

Animal MRI cradles are specially designed to position the animal during scanning. Having a dedicated setup reduces positioning variabilities within and across subjects. A tooth bar and/or ear bars can ensure proper fixation of the head of the animal and reduce motion. Imaging of awake animals can also be achieved via an integrated body restraint. For imaging awake NHPs, head fixation can be achieved using non-invasive helmets or invasive head post implantation^33^. The animal cradle can supply volatile anesthetics (e.g., isoflurane), injectable anesthetics (e.g., medetomidine), or a combination of anesthetics, which is now standard for rodent fMRI^10^. To control the depth and effects of anesthesia and to maintain the well-being of the animal while in the bore, respiratory rate and body temperature are monitored.

Additional equipment for sensory or optogenetic, sonogenetic, or pharmacological stimulation of the animal under study requires the placement of additional fibers to be attached to the animal’s skull. Complementary measurements, e.g., electrophysiology, optical, optoacoustic, or ultrasound imaging, or cameras and tubes for reward delivery in awake behaving, animals require installation of additional equipment (Fig. 3)^33^.

### Optimizing EPI parameters for the spatiotemporal resolved mapping of functional brain states

Image quality as well as the achievable spatial and temporal resolution depends not only on the hardware used, but also on the protocol parameters. Parameters for EPI protocol optimization are summarized in Table 1.

**Table 1:** Key parameters for optimizing EPI protocols

Parameter	Typical values	Impact on study design and image quality	Comments
Image geometry			
Number of slices	1–10	More slices increase gradient duty and decreases TR.	Use minimum number of slices to cover the area of interest and to achieve desired spatial resolution.
Slice gaps	10–20%	Reduce interferences between neighboring slices	Slice gaps may not be desired for whole brain coverage.
Slice acquisition order	Sequential	Possible cross-talk between neighboring slices.	Use slice gaps, if possible, to avoid cross-talk.
	Interleaved	Susceptible to through-plane motion that creates “spin-history” artifact.	
Slice thickness	Mouse: 0.3–0.8 mm Rat: 0.4–1.0 mm Marmoset 0.5–1.2 mm	Thinner slices lead to less image distortions and signal dropouts, but decrease SNR, increase gradient duty and acoustic noise. It needs also more slices to cover an area and thus affects temporal resolution.	Less phase dispersion within a slice. Higher fields mitigate SNR loss.'''
Spatial resolution	Mouse: ~ 200 µm Rat: ~200–400 µm Marmoset: ~500 µm	Higher spatial resolution for higher anatomical specificity. But increasing the matrix size reduces SNR, increases gradient duty and decreases temporal resolution (i.e. increasing the number of phase-encoding steps).	As low as possible for enough SNR and high TR. Achievable spatial resolution depends on hardware. No need to have the same image size in read/phase.
Image contrast			
TE	3T: ~30 ms; 4.7T: ~25 ms, 7T: ~18–25 ms; 9.4T: ~14–18 ms; 11.7: ~10–15 ms; 15.2T: ~11 ms	A shorter TE results in reduced image distortion but also in reduced BOLD changes ($${fCNR})$$, while a longer TE decreases the baseline SNR due to decay of the transverse magnetization.	Optimal TE depends on magnetic field strengths.
TR	1000–2000 ms Ultrafast < 1000 ms	TR is limited by the geometry (see above). Decreasing TR increases temporal resolution but also increases gradient duty.	Adapt flip angle accordingly.
Bandwidth	150–400 kHz	Increasing bandwidth reduces images distortions and allows to reduce TE. But increasing the bandwidth reduces SNR and increases the gradient duty cycle, gradient heating and acoustic noise.	Keep as low as possible within the acceptable distortion and temporal resolution. May overall be limited by the gradient performance.
Segments	Normally 1 (single-shot), 2–4 (multi-shot)	Segmenting allows to reduce TE with less time for T2* decay and reduces distortion. It also reduced gradient duty. Drawbacks are an increase in measurement time (proportional to the number of segments), resulting segmentation artifacts from misalignments of the multiple acquired segments, and motion sensitivity.	Segmentation artifacts can be mitigated by using a navigator.
Acceleration			
Partial Fourier	Up to ~1.4	The technique can be used to increase the spatial and temporal resolution and coverage and to reduce gradient duty (especially when the matrix size is relatively large). Also, a shorter TE can be achieved. Use of partial Fourier leads to smoothing and a reduction in SNR and ${tSNR}$.	Higher Partial Fourier may result in artifacts. Reduction in SNR can be offset by SNR gains when reducing TE.
GRAPPA	Maximum acceleration factor = to half of active coil elements.	Use of GRAPPA to decrease TR and/or increase spatial resolution and/or coverage. Allows to reduce TE with less image distortion. Reduces SNR and may have reconstruction artifacts with higher acceleration factors.	Maximum acceleration factor depends also on the arrangement of coil elements. Use only when a higher spatial and temporal resolution is required than can be achieved with a full k-space EPI acquisition.
Simultaneous multi-slice		Reduces scan time. Can be used to reduce TR, to have higher spatial resolution and/or greater slice coverage (e.g., when thinner slices are used), and to decrease gradient duty. Does not suffer from SNR penalty. May come with reconstruction artifacts.	Requires a multi-channel coil for signal reception. Scan time reduction is proportional to slice acceleration factor. Radiofrequency tissue heating is a potential limitation.

**Fig. 4:**
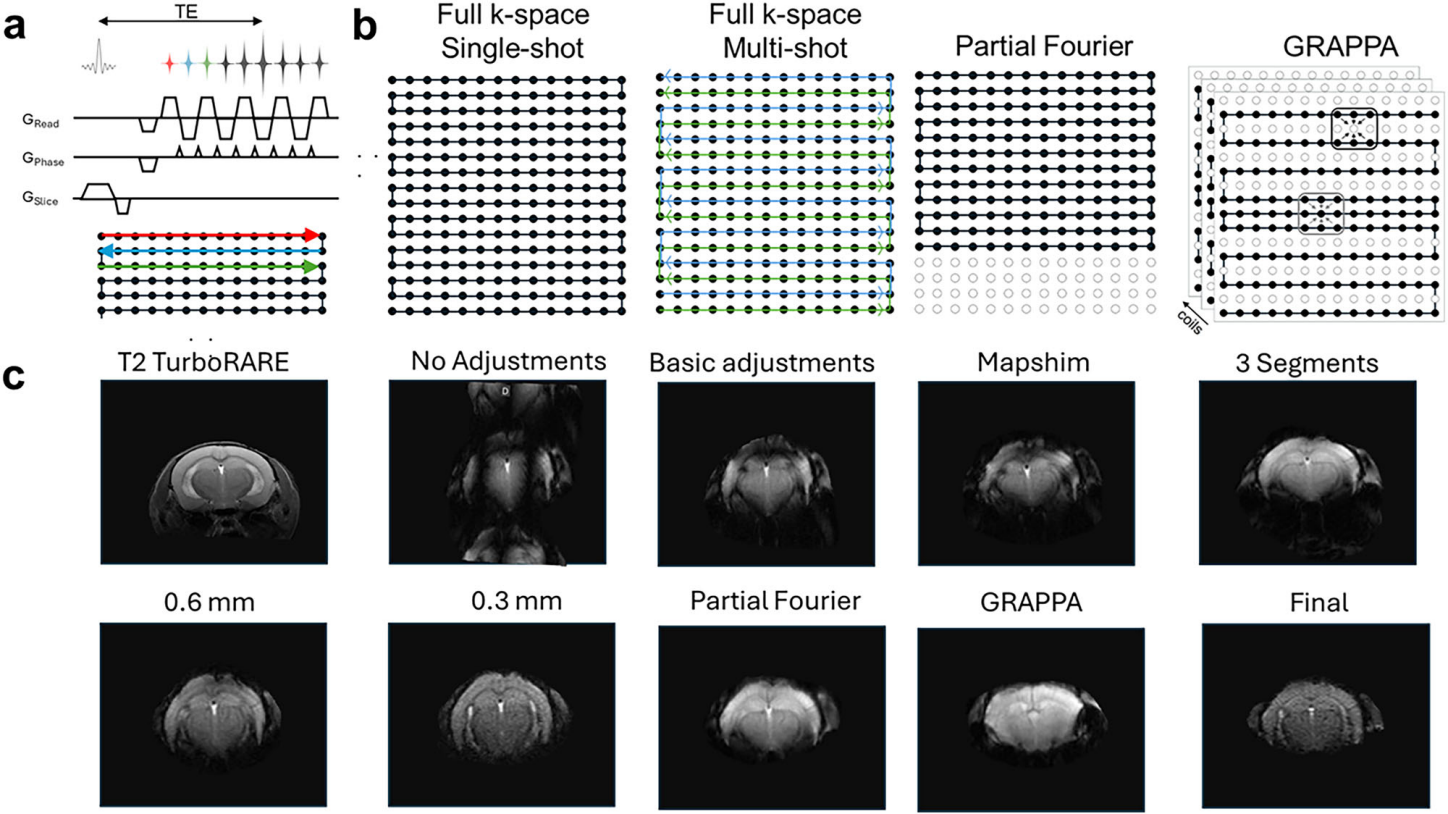
Parameters for EPI. **a** Pulse sequence diagram for a GE EPI. Gradient (G) shapes are shown for the read, phase and slice direction. **b** k-space trajectories and sampling for different EPI sequences. In single-shot EPI all k-space lines are sampled after a single excitation pulse. Frequency-encoding is along horizontal axis while phase-encoding is along the vertical. In multi-shot EPI parts of k-space are sampled with each shot. Partial Fourier reduces the number of phase-encoding steps by a one-sided truncation. This accelerates EPI acquisition. GeneRalized Autocalibrating Partially Parallel Acquisitions (GRAPPA) can be used when a multi-channel coil is used for signal reception. A reduced data set in the phase encoding direction of k-space is acquired, significantly reducing the acquisition time. **c** Effects of different image parameters on EPI image quality. Shown are axial slices of a mouse brain, with a GE EPI basic protocol without and with adjustments (study shim, ghost correction, receiver gain with trajectory measurement) and using fieldmap-based shimming, segmentations, thinner slices, and acceleration techniques. For the final image, a single-shot EPI with 0.3 mm slices, using partial Fourier and mapshim, was combined in the acquisition protocol. A T_2_-weighted fast spin echo (Rapid Acquisition with Relaxation Enhancement (RARE) was acquired as an anatomical reference. Data was acquired for educational purposes using a BioSpec Maxwell 94/17. All Bruker in vivo animal work was approved by the institutional animal care and use committee (IACUC) and local authorities and conducted under a valid study permit (Germany AZ 123456).

In EPI, k-space is sampled by fast switching bi-polar frequency-encoding gradients interspersed by short gradient pulses (blip pulse) in the phase-encoding direction (Fig. 4). To maximize speed, data is often sampled during ramp up/down of the readout gradients and therefore requires a prior k-space trajectory measurement, which is used to redistribute data in k-space during reconstruction (regridding,) thus preventing misalignment artifacts. Since adjacent k-space lines are acquired in opposite directions, they need to be time-reversed during reconstruction. Phase errors between adjacent lines cause Nyquist N/2 ghost artifacts. This can be corrected in the reconstruction using information acquired during the receiver gain adjustment.

An important consideration for EPI protocols is the image geometry. Parameter choice depends on the size of the animal, the brain volume that needs to be covered, and the desired spatial and temporal resolutions. The number of slices should cover the area(s) of interest, but should be set at the minimum (even single slice) to reduce repetition time (TR)^34,35^. Typical values for slice thickness and image size are given in Table 1. Slice thickness is a parameter that greatly affects EPI image quality as a reduction leads to less phase dispersion within a slice and reduces distortions and signal dropouts (Fig. 4). However, reducing slice thickness also decreases SNR and ${tSNR}$ and increases gradient duty cycles, especially if more slices are acquired at a given time to attain tissue coverage. Thinner slices may also require increasing the number of slices to cover the volume of interest, which may reduce temporal resolution and further increase the gradient duty cycle. Adding slice gaps is recommended as it reduces interferences between neighboring slices for sequential acquisitions. Alternatively, interlaced acquisition can be used without gaps. While high spatial resolution in fMRI is often desired, increasing the image size increases gradient duty. Furthermore, increasing the image size in the phase direction increases TR and thus reduces temporal resolution.

### Parameters for optimizing EPI image quality

Several interrelated protocol parameters influence EPI image quality. Optimizing echo time (TE) is vital for ${fCNR}$, since it influences both the magnitude of $\frac{\triangle S}{S}$ and the baseline SNR (see typical values in Table 1). While a shorter TE results in reduced $\frac{\triangle S}{S}$, a longer TE decreases SNR due to decay of the transverse magnetization and results in increased image distortions. Shorter TR increases temporal resolution while increasing gradient duty. In addition, a shorter TR reduces SNR due to the use of lower flip angles.

In EPI, image distortion occurs when the B_0_ offset at the pixel is greater than its pixel bandwidth. The distortion is observed in the phase encoding direction because the bandwidth in phase encoding direction (Eq. (2)) is smaller compared to the bandwidth in the readout direction (Eq. (3)).

2 $${{bandwidth}}_{{phase}}=\frac{1}{{echo\; spacing}}=\frac{{acqusition\; matrix}}{{readout\; matrix\; size}}$$

3 $${{bandwidth}}_{{read}}=\frac{1}{{dwell\; time}}={acquisition\; bandwidth}$$

The bandwidth can be increased to reduce distortions also allowing a reduction of TE. But increasing the bandwidth reduces SNR and increases the gradient duty cycle, gradient heating and acoustic noise and may overall be limited by the gradient performance.

Usually, BOLD fMRI experiments are performed with single-shot EPI, where all k-space lines are sampled after a single excitation pulse^36^. However, multi-shot EPI (i.e., segmenting) can be performed where only parts of k-space are sampled with each shot (Fig. 4). Segmenting allows to reduce TE with less time for T_2_* decay and reduces distortion, but one drawback is that the total measurement time increases proportional to the number of segments. An additional disadvantage is that segmentation artifacts can result from misalignments of the multiple acquired segments, which can be mitigated by using a navigator. Furthermore, multi-shot EPIs are sensitive to the motion of the animal.

EPI is very sensitive to magnetic field inhomogeneity (Fig. 4). When introducing an animal into the magnet, macroscopic field inhomogeneities arise that are caused by the differences in magnetic susceptibility in areas of heterogeneous tissues (e.g., gray and white matter) or at air-tissue interfaces, which scale with the applied B_0_. Local B_0_ variations result in intravoxel dephasing and signal loss, and the frequency-encoding in EPI causes an additional pixel shifting, leading to both signal distortion and loss.

Active shimming is achieved via specialized shim coils that are used to produce spherical harmonic functions to homogenize the magnetic field within the brain or a brain region. In fieldmap-based shimming, a low-resolution magnetic fieldmap of the head region is first acquired. Fieldmapping can, for example, be performed by collecting GE images at differing TEs, and then by calculating the field from the phase difference between the images^37^. A shim volume can then be defined by automatic segmentation procedures^38^, or manual placement over a brain region^39^, after which the magnetic field will be homogenized by setting higher order shims. For ultrahigh field systems and/or when the target area is difficult to shim (e.g., amygdala, entorhinal cortex), the use of spin echo EPI should be considered as it is more robust against susceptibility artifacts. Moreover, the spin echo BOLD contrast is less sensitive to macrovessel signal contributions than GE EPI is^40^.

An alternative approach is to collect images with blips in opposite phase directions and then reconstruct an undistorted image^41^. The EPI image is compressed or stretched where the homogeneity of B_0_ is worse than the pixel bandwidth in the phase direction. By reversing the phase encoding direction, the direction of the geometric distortion in the image is flipped, and a pair of forward and reversed phase-encoded images can be used to correct the image distortion. It should be noted that acquiring two sets of images with opposing blips doubles the acquisition time, and the choice of phase encoding directions can affect fMRI statistics, and thus care should be taken when making this selection^42^.

### Making fMRI acquisitions faster

Several techniques can be used to accelerate EPI. While acceleration might be beneficial for some parameters, the effect on others needs to be considered.

In Partial Fourier imaging, the number of phase encoding steps is reduced by a one-sided truncation (Fig. 4). Rather than acquiring every single echo in the EPI echo train, only just over half of the echoes are acquired by omitting the first, i.e., one quarter of the phase-encoded echoes in the train. The technique can be used to increase spatial and temporal resolution and coverage. It is also useful to reduce gradient duty, especially when the matrix size is relatively large. Moreover, since the number of k-space lines needed to reach the center of k-space is reduced, a shorter TE can be achieved. Use of partial Fourier leads to smoothing and a reduction in SNR and ${tSNR}$.

If a multi-channel coil is used for signal reception, the GeneRalized Autocalibrating Partially Parallel Acquisitions (GRAPPA) technique can be used (Fig. 4). A reduced data set in the phase encoding direction of k-space is acquired. The maximum acceleration factor is equal to half of the number of active coil elements, but it also depends on their arrangement. The missing data points in the sub-sampled k-space data are calculated from the acquired signals from all coil elements using a predetermined interpolation kernel. The weights of the kernel are determined using a separate calibration scan consisting of a low-resolution, fully sampled k-space data set. By skipping k-space lines during signal acquisition in GRAPPA, the minimum TR is reduced, and this can be used to improve the temporal resolution of the EPI scan. Conversely, the savings in sampling time can be used to image a larger matrix to obtain a higher in-plane resolution or to increase the number of slices to obtain a wider through-plane coverage. GRAPPA can also be used to decrease TE, which in turn can reduce image distortions. However, using GRAPPA reduces SNR. It can also lead to reconstruction artifacts, which tend to increase with increasing acceleration factors. Use of GRAPPA makes the acquisition more motion sensitive because of the necessity of performing calibration scans. Thus, GRAPPA should only be used when a higher spatial or temporal resolution is required than can be achieved with a full k-space EPI acquisition.

An alternative approach to undersampling techniques is simultaneous multi-slice EPI, also known as multiband EPI^43^. It allows the simultaneous acquisition of multiple slices in the slice direction. Like GRAPPA, multiband EPI also requires a multi-channel coil for signal reception. It reduces scan time as a factor of slice acceleration. It can be used to reduce TR, to have higher spatial resolution and/or greater slice coverage (e.g., when thinner slices are used), and to decrease gradient duty. Unlike partial Fourier and parallel imaging, simultaneous multi-slice EPI does neither rely on the omission of k-space samples nor the reduction of the echo train length and thus does not suffer from the SNR penalty associated with these techniques. However, simultaneous multi-slice EPI may come with reconstruction artifacts. Moreover, radiofrequency tissue heating is a potential limitation of simultaneous multi-slice EPI as the peak power of a multiband radiofrequency pulse scales with the square of the number of simultaneously excited slices. This is more problematic for spin echo than GE EPI sequences.

Acceleration techniques can be combined with each other in an EPI protocol, often with a low number of slices to reduce TR and thus to achieve ultrafast fMRI with subsecond resolution^34,35^. Moreover, radial encoding has been introduced recently for ultrafast fMRI acquisition^44^.

### Adapting EPI for experimental paradigms

BOLD signals can be measured from task-free conditions (resting-state) or from stimulus-evoked conditions. The data acquisition can be synchronized with the stimulation. For fMRI experiments, EPI data is repetitively collected based on the study design. The use of dummy scans with repetitions is recommended for stabilization of the MRI signal. It is also advisable to activate drift compensation^45^. As EPIs run often at very high gradient duty cycles for prolonged periods of time to obtain images with high temporal and spatial resolution, the resulting heating of the gradient coils and amplifiers may cause a drift of the resonance frequency and thus reduce ${tSNR}$^46^. When a drift compensation functionality is enabled, a Free Induction Decay signal from the entire subject is acquired with a small flip angle (~1 degree) at the end of each repetition, and the amount of frequency drift is calculated from the phase difference between successive signals.

### Expert consensus protocol

Recent rodent resting-state fMRI multi-center studies have examined the effect of using different hardware configurations (field strengths, RF coils, etc.), acquisition protocols, and experimental conditions (e.g., anesthesia) on fMRI read-outs^9,11^. Based on the expert consensus of one initiative, rat fMRI acquisition protocols (StandardRat) were introduced, providing imaging parameters for scanners at different field strengths^11^.

### Preprocessing steps to improve fMRI data quality

Data analysis of BOLD fMRI is challenging as the signal changes are relatively low and the data exhibits a complex noise structure. Prior to statistical analysis, fMRI data typically undergoes a series of preprocessing steps aimed at removing artifacts, reducing noise, and validating model assumptions^47,48^. A first step is motion correction, as animal motion during scanning prevents the precise spatial correspondence between voxels and anatomical areas over time. While images with large motion artifacts may be excluded, minor motion can be corrected. Another step is slice timing correction. Since EPI data of multiple slices is acquired sequentially, there is an intrinsic delay between slice acquisition times. Slice timing correction adjusts the time course of voxel data in each slice to account for these differences by interpolating the information in each slice to match the timing of a reference slice. In addition, spatial transformation is performed to either co-register the acquired EPI images of an animal with those acquired from a different animal or a structural MR image. Alternatively, a normalization procedure is applied to warp EPI images to a standard template brain for group analysis. The spatial transformation can be complemented by removing non-brain areas from the analysis, such as skull stripping or masking.

Spatial smoothing/filtering is another step during which data points are averaged with their neighbors, suppressing high-frequency signals while enhancing low-frequency ones. This step increases the SNR but reduces the effective spatial resolution. Furthermore, it ensures that the assumptions of random field theory, commonly used to correct for multiple comparisons, are valid. Similarly, temporal filtering can increase the SNR of the measured BOLD signal by removing the effects of confounding signals from slow scanner drifts and cardiac and respiratory signal fluctuations.

### Data analysis tools for generating activation and connectivity maps

There are many computational tools available for the analysis of data from different experimental paradigms^9,11,47^. Analysis of stimulus-evoked BOLD responses aims to identify brain areas that are significantly activated when a stimulus condition is applied. The result is an activation map that shows the degree of statistical significance with which each pixel can be activated. The most widely used approach for the analysis of stimulus-evoked fMRI data is based on a general linear model^49^. The model uses the convolution of the hemodynamic response function with the stimulus paradigm. However, since the hemodynamic-response function differs across species and brain regions^50,51^, and is influenced by experimental factors like anesthesia, a canonical hemodynamic response function cannot be used. A data-specific function needs to be applied instead. To make inferences at the group-level, the general linear model can be used to model the time series of the BOLD signal as a linear combination of different signal components and to test whether the activity in a defined brain region is systematically associated with a particular condition of interest.

The aim of the analysis of resting-state fMRI data is to estimate correlations between different brain regions. These correlations may indicate a functional connectivity between those regions. The first model-driven computational method applied to resting-state fMRI was seed-based correlation analysis. The method is based on the activity in an a priori defined seed region, which may be a volume or a single voxel, which is compared to that in all other voxels in the brain. To overcome the inherent limitations of model-based analyses, exploratory data-driven methods, which require neither prior information nor a previously defined model, have been applied to fMRI data. These methods include principal component analysis, independent component analysis, dynamic functional connectivity analysis, and tools from graph theory.

### Applications of preclinical fMRI in brain studies

To understand normal brain function and its correlation to behavior, it is essential to decipher the underlying cellular signaling. Mapping of brain function under controlled experimental conditions and using interventional tools for manipulating neuronal and non-neuronal signal transduction in a cell-type-specific or cell population-specific manner is only feasible in preclinical studies.

**Fig. 5:**
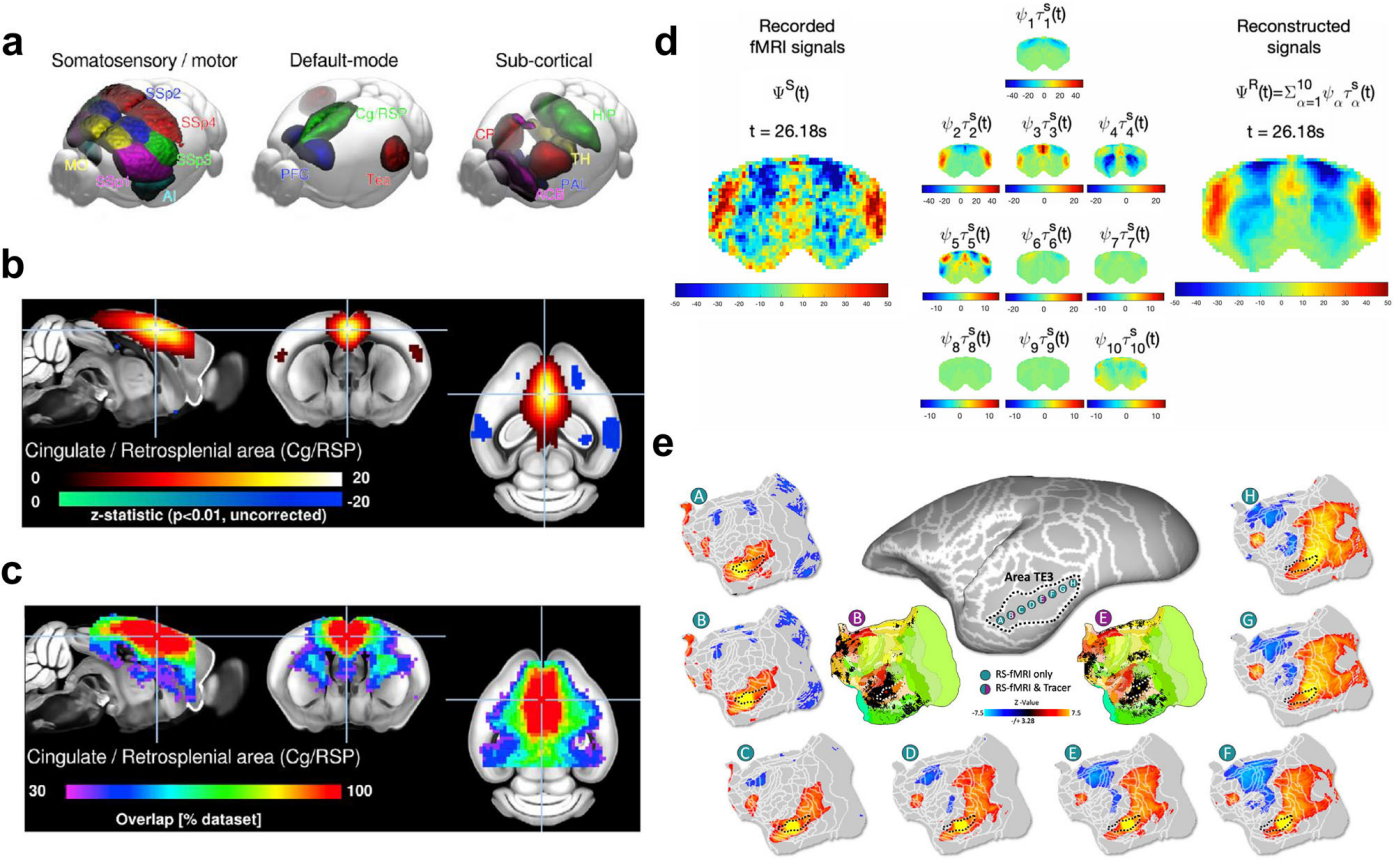
Functional connectivity in the brain of different species. **a**–**c** Functional networks in the mouse brain. Group-level independent component analysis estimated across 98/255 “specific functional connectivity” scans reveals canonical mouse components. **a** All components presented a marked bilateral organization. Nine components were found to overlap principally with the isocortex including regions attributed to latero-cortical and salience network, and default-mode network by seed-based analyses, three components overlapped with the striatum, one with the hippocampal areas, and one with the thalamus. Detailed representations of the cingulate/retrosplenial area component (Cg/RSP). **b** One-sample *t* tests within datasets indicate that 100% of datasets presented significant functional connectivity (*p* < 0.05, uncorrected) within the Cg/RSP component. **c** Functional connectivity relative to Cg/RSP is found specifically in the anterior cingulate area but not in the primary somatosensory in 79% of the individual scans following dual regression. Taken from [Grandjean 2020]^9^. **d** Functional connectivity in the rat brain. Principal component analysis of ultrafast fMRI signals (temporal resolution = 38 ms). (Left) fMRI signals in *n* = 1463 brain voxels band-pass filtered between 0.01 and 0.3 Hz recorded from a representative rat under medetomidine only. (Middle) Each of the 10 spatially defined principal modes of covariance is scaled over time by its corresponding temporal signature in scan S to illustrate the standing wave dynamics. (Right) The signals recorded in scan S are reconstructed as the linear sum of the 10 principal components multiplied by their corresponding temporal signature in scan S. To account for differences in power across components, color bar limits are set to ±4 standard deviations of the corresponding temporal signatures. Taken from [Cabral 2023]^35^. **e** Voxel-wise comparison of functional brain connectivity of a marmoset (green, from marmosetbrainconnectome.org) with existing tracer-based resources (purple, from marmosetbrain.org) in the area TE3. Taken from [Schaeffer 2022]^54^.

Resting-state fMRI has been used to outline the functional connectivity of the brain in different species (Fig. 5). It has been shown that the rodent brain is organized in large-scale networks, like those reported in humans^9,35^. Studies of rodents of different ages have shown network-level changes during development^32^, and aging^52^. Investigations in marmosets revealed that NHPs have a similar functional connectivity of the brain to rodents, but also show some species-specific peculiarities^53^. Ultrafast fMRI has enabled advanced analyses of the temporal interactions between brain regions at neuronally relevant scales^35^. Functional connectivity has been compared with tracer-based resources to investigate the cellular underpinnings of the connectome^54^.

Stimulus-evoked fMRI allows for the investigation of neural activity as a response to a specific stimulus or task. This has been used to map the functional BOLD response to somatosensory^30,34,55^, auditory^30^, visual^56^, and olfactory^30^ evoked responses in awake and anesthetized rodents and NHPs. In addition, pharmacological MRI has been used to assess the mechanisms of action of neurobiologically active drugs as well as to investigate specific neurotransmitter systems with BOLD contrast^57^.

**Fig. 6:**
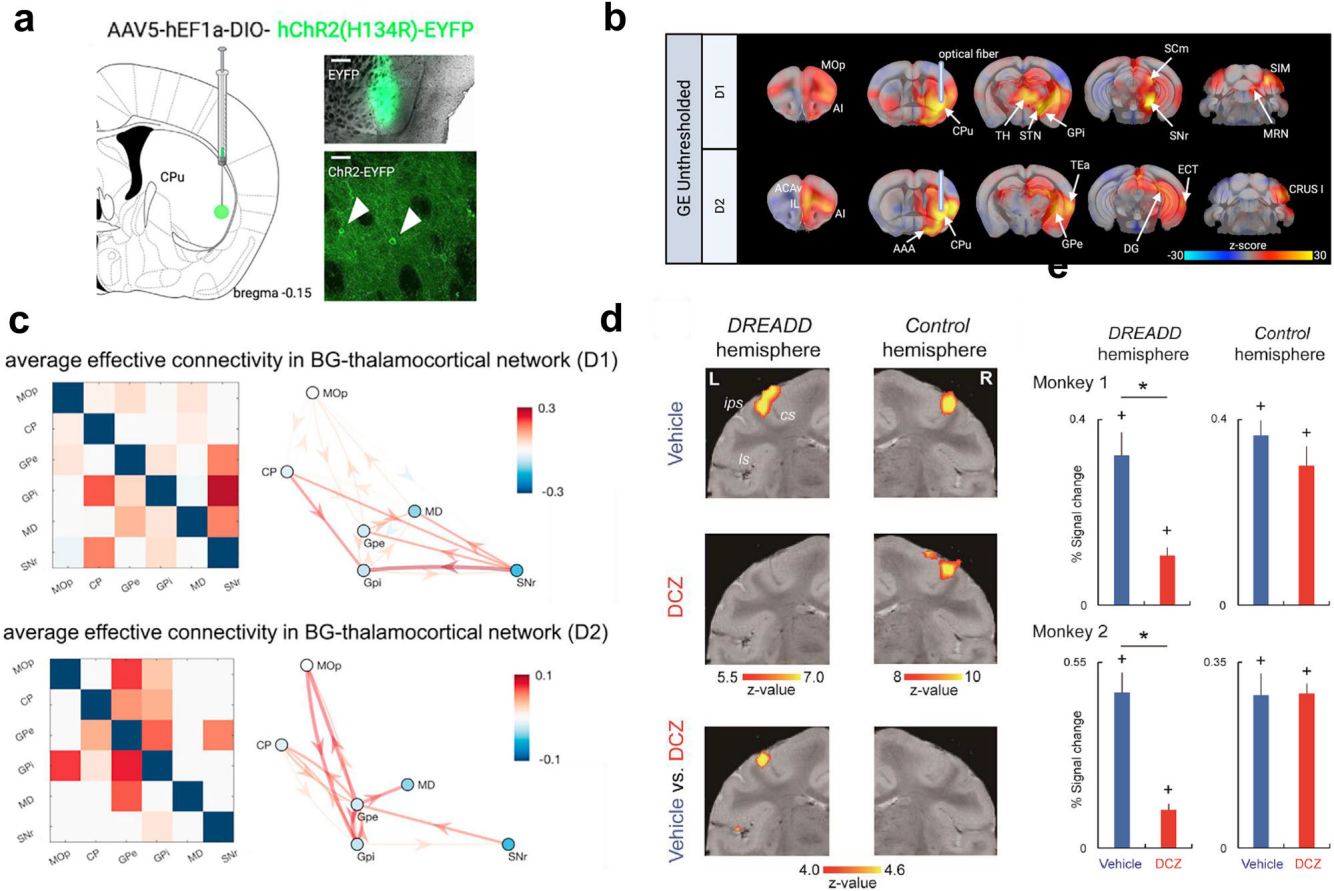
Studying brain function with optogenetic and chemogenetic activation. **a**–**c** Optogenetic activation of striatal cells expressing D1 (D1R) and D2 (D2R) dopamine receptors in the mouse. **a** A virus carrying an excitatory opsin was injected into the right ventrolateral caudate putamen of D1- and D2-Cre mice. Viral expression was confirmed histologically. **b** Unthresholded generalized linear model z-stat activation maps of D1R and D2R cell stimulation. **c** Average connectivity pattern in the basal ganglia (BG)-thalamocortical network during D1R cell stimulation and during D2R cell stimulation. Striatal D1R/D2R stimulation evokes distinct activity within the (BG)-thalamocortical network and differentially engages cerebellar and prefrontal regions. Primary motor cortex (MOp), caudate putamen (CP), external globus pallidus (GPe), internal globus pallidus (GPi), mediodorsal nucleus of thalamus (MD), substantia nigra (SNr). Taken from [Grimm 2021]^58^. **d**, **e** Chemogenetic-induced silencing in the macaque brain leads to attenuation of the sensory-evoked BOLD signal in the unilateral hand index finger region. **d** Primary somatosensory cortex activation in monkey 1 evoked by cutaneous tactile stimulation of the index finger of each hand contralateral (left) and ipsilateral (right) to the designer receptor exclusively activated by designer drug (DREADD)-expressing hemisphere. Activation maps of the primary somatosensory cortex for vehicle and deschloroclozapine (DCZ), and the difference between them. **e** Percent signal change calculated for primary somatosensory cortex ROIs in the contralateral hemisphere to the stimulated hand for each monkey. Results for DREADD-expressing (left) and the opposite (right) hemispheres are depicted. Blue and red columns depict the results in vehicle and DCZ conditions, respectively. Chemogenetic inhibition also led to impaired fine grasping with the contralateral hand. Error bars, SEM. *p < 0.001, unpaired *t* test, corrected for multiple comparisons. +p < 0.001, paired *t* test for difference from baseline, corrected for multiple comparisons. Taken from [Hirabayashi 2021]^59^.

Chemogenetic and optogenetic excitation has been combined with fMRI to elucidate the roles of specific functional downstream regions in global brain functioning^4–7,58–60^; to evaluate mechanistic models of brain function^6^; and to examine the network-level mechanisms that underlie the role of specific brain regions in certain behavioral states (Fig. 6)^4,58,59^. Moreover, the combination of fMRI with electrophysiological measurements and immunohistochemistry have provided multiplex data for functional analysis^7^.

Both stimulus-evoked and resting-state functional networks involve brain regions that are hierarchically yet reciprocally connected by intracerebral circuits. Approaches to successfully dissect local and long-range fMRI responses at the circuit level have been proposed, which use targeted silencing of specific brain regions with optogenetic or sensory stimulation to suppress downstream networks^7,59–62^. Inhibiting one brain region under resting conditions inevitably suppresses excitatory output to downstream signaling pathways. The downregulated neuronal activity reflects the degree of interregional communication under basal conditions. This can be applied for mapping long-range circuits across the whole brain, complementing traditional anatomical tracing studies. Similarly, local silencing during exposure to external stimuli suppresses downstream activity without compromising the upstream and/or collateral inputs from other brain regions. Thus, downstream circuit contributions to stimulus-evoked fMRI can be determined by comparing evoked BOLD responses with and without focal inactivation. These approaches have been applied to study sensory processing^59,61^, and the role of a cortical node^7^; to relate to behavioral effects^59,62^, as well as to investigate functional reorganization caused by chronic pain^62^.

The underlying principles of neurovascular coupling are still not well understood, and thus, the interpretation of BOLD maps is not straightforward. Preclinical studies offer the ability to combine fMRI with electrophysiological and optical read-outs such as wide-field fluorescence, fiber photometry, and two-photon microscopy with fluorescent indicators that can probe activity of neuronal cells^63,64^. These helped to decipher the neuronal and non-neuronal contributions to the BOLD signal. Complementary preclinical investigations using intrinsic signal optical imaging and two-photon microscopy have been used to investigate the spatial and temporal properties of neurovascular coupling in health and disease and thus inform models for BOLD-based fMRI measurements^65,66^.

Preclinical fMRI studies in models of human neurological and psychiatric disorders have been instrumental for investigating how changes at the molecular and cellular level relate to alterations in brain function and circuitry, and disease symptomatology. For example, traumatic and ischemic brain injuries and inflammation lead to adaptive neuroplastic alterations^67–69^.

**Fig. 7:**
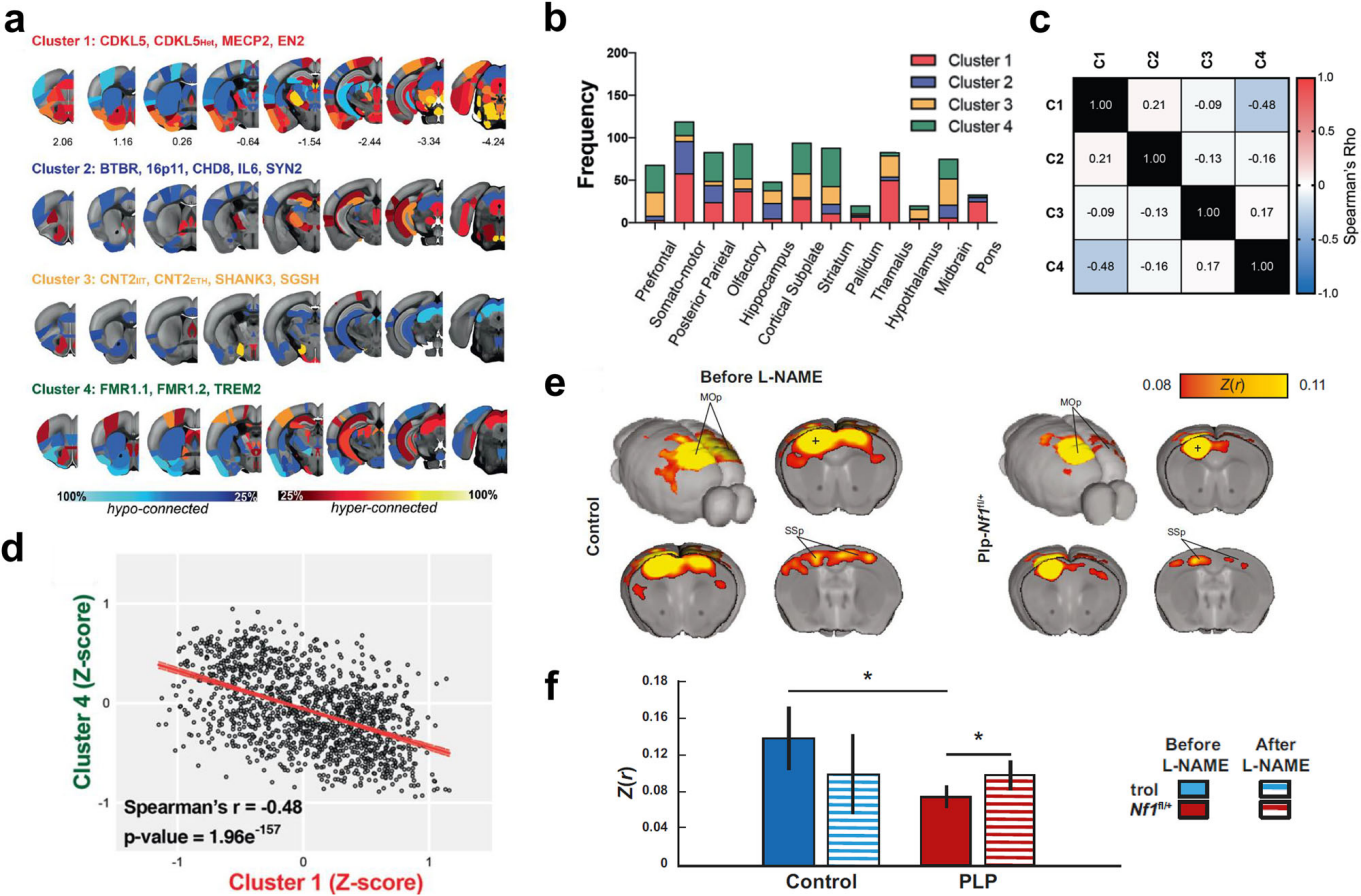
fMRI in animal models of psychiatric and neurological disorder. **a**–**d** Functional connectivity deficits in mouse models of autism. The mapping reveals a spectrum of four functional connectivity subtypes. **a** Rendering of regional connectivity deficits in the four clusters at the node level, revealing a heterogeneous set of brain areas with prominent over- and under-connectivity. Data are visualized in Allen Mouse reference space. **b** Number of connections (displayed as stacked frequencies) that exhibited abnormalities at the parent level. **c** Correlation matrix between all clusters, considering all 545 edges. **d** A significant negative correlation was found between Cluster 1 and Cluster 4. Spearman’s rho = −0.48, *p* = 1.96e^-157^. Taken from [Zerbi 2021]^72^. **e**, **f** Therapeutic rescue of reduced functional connectivity in a mouse model of neurofibromatosis type 1. **e** Group average correlation maps between control and mice with oligodendrocyte-specific Nf1 deletion (Plp-Nf1^fl/+^) demonstrate reduced interhemispheric connectivity in Plp-Nf1^fl/+^ mice. Z(r) Fisher’s Z-transformed r, MOp primary motor cortex, SSp primary somatosensory cortex. **f** Rescue of connectivity in Plp-Nf1^fl/+^ mice by inhibition of nitric oxide synthase. Correlation of seed-to-seed analysis in the primary motor cortex (denoted as a plus sign in the maps shown in **e**, plotted for each group before and after L-arginine methyl ester (L-NAME) treatment. Taken from Asleh^75^.

Many neurological and psychiatric disorders are of genetic etiology. The ability to engineer the genome of animal species used in fMRI research offers a route to simulate such disorders allowing a systematic study of pathological changes (Fig. 7). To this end, resting-state fMRI studies have been applied in numerous disease conditions, including Alzheimer’s disease^2^, schizophrenia^3^, anxiety^70^, pain^71^, and autism^72–74^, and to monitor the effect of novel treatments^75,76^. An interesting approach is to use stimulus-evoked fMRI to assess the sensory sensitivity of specific brain regions and to relate it to behavioral states in transgenic models of brain diseases^74^.

## Conclusion

Preclinical BOLD fMRI is a fast-evolving field aimed at non-invasively studying brain function. To date, most data is acquired with T_2_*-weighted GE EPI which puts high demands on SNR. With the progress in preclinical MRI hardware, in particular the use of higher and ultrahigh field systems and the construction of cryogenic radiofrequency coils, and EPI protocols, and by carefully controlling the physiology of the animal under study it has been possible to collect BOLD responses in a variety of animal species and experimental conditions with high temporal and spatial resolution. The application of the technique to genetically engineered animals and the combination with interventional tools, with omics and histological tissue read-outs has extended our understanding of brain function beyond the reach of human research studies and holds promising implications for future studies.

## Data Availability

No datasets were generated or analyzed during the current study.
